# Dissecting the causal association between social or physical inactivity and depression: a bidirectional two-sample Mendelian Randomization study

**DOI:** 10.1038/s41398-023-02492-5

**Published:** 2023-06-08

**Authors:** Guorui Zhao, Zhe Lu, Yaoyao Sun, Zhewei Kang, Xiaoyang Feng, Yundan Liao, Junyuan Sun, Yuyanan Zhang, Yu Huang, Weihua Yue

**Affiliations:** 1 Peking University Sixth Hospital, Peking University Institute of Mental Health, Beijing, 100191 China; 2 National Clinical Research Center for Mental Disorders (Peking University Sixth Hospital), Beijing, 100191 China; 3 NHC Key Laboratory of Mental Health (Peking University), Beijing, 100191 China; 4 National Engineering Research Center for Software Engineering, Peking University, Beijing, 100871 China; 5 PKU-IDG/McGovern Institute for Brain Research, Peking University, Beijing, 100871 China; 6 Chinese Institute for Brain Research, Beijing, 102206 China

**Keywords:** Genomics, Depression

## Abstract

A growing body of research suggests that social or physical activity can affect the risk of Major depressive disorder (MDD). However, the bidirectional relationship between them remains to be clarified further, especially between inactivity and MDD. Here, we performed a two-sample Mendelian Randomization analysis using genetic variants associated with social/physical activities and MDD, and assessed the mediating effect of obesity-related measures and brain imaging phenotypes. The dataset on MDD, social activities, and physical activities included 500,199; 461,369; 460,376 individuals, respectively. Information regarding body mass index (BMI), body fat percentage (BFP), IDPs for 454,633; 461,460; 8,428 participants, respectively. We identified bidirectional causal relationships between sport clubs or gyms, strenuous sports, heavy do-it-youself, other exercises and MDD. We also observed that leisure/social inactivity (odds ratio [OR] = 1.64; *P* = 5.14 × 10^−5^) or physical inactivity (OR = 3.67; *P* = 1.99 × 10^−5^) caused an increased risk of MDD, which were partially mediated by BMI or BFP and masked by the weighted-mean orientation dispersion index of left acoustic radiation or volume of right caudate. Furthermore, we discovered that MDD increased the risk of leisure/social inactivity (OR = 1.03; *P* = 9.89 × 10^−4^) or physical inactivity (OR = 1.01; *P* = 7.96 × 10^−4^). In conclusions, we found that social/physical activities reduced the risk of MDD, while MDD in turn hindered social/physical activities. Inactivity may increase the risk of MDD, which was mediated or masked by brain imaging phenotypes. These results help to understand the manifestations of MDD and provide evidence and direction for the advancement of intervention and prevention.

## Introduction

Major depressive disorder (MDD) is a common medical condition that severely lowers the quality of life and limits psychosocial functioning [1]. MDD is a leading cause of disability and plays an important role in the global burden of disease [2]. The etiology of MDD includes both physiological and social factors. In contrast with factors that increase MDD risk such as poverty, unemployment, family history, illness, and life events [2, 3], leisure/social activities and physical activities have received increasing attention as relatively easy-to-control risk factors [4–6].

Meta-analyses of prospective cohort studies showed that physical activities are associated with a reduced risk of MDD, regardless of age and geographical region [5, 7]. Other studies have indicated that leisure activities or good social relationships are related to lower depressive symptoms or a lower prevalence of MDD [8–10]. Symptoms of MDD can also include loss of energy and interest in previously enjoyed activities, which may lead to decreased social or physical participation [11]. This provides evidence of a bidirectional causal relationship between social/physical activity and MDD which could not be ascertained through traditional observational studies [6, 12]. Considering that physical activity is often related to the prevention and management of obesity [13, 14], obesity traits could be mediators of the effect of activity on MDD.

Mendelian Randomization (MR) is an epidemiological method used to infer possible causal relationships between exposure and outcome by introducing single nucleotide polymorphisms (SNPs) associated with exposure (such as physical activity) as instrumental variables (IVs) [15]. As alleles follow the principle of random segregating combinations, the causal effect is theoretically unperturbed by confounding factors and reverse causes [16]. IVs can be extracted from publicly available genome-wide association studies (GWAS) summary statistics, which are widely used for two-sample MR [17]. A previous MR study showed a protective relationship between accelerometer-based activity and MDD [6]. An exposure-wide MR analysis assessed bidirectional causality between more than 100 modifiable factors (including those of social and physical activity) and MDD [18]. However, physical activity GWAS could include inactivity, and the relationship between inactivity and MDD remains unexplored. Guo et al. showed a causal relationship between brain phenotype and psychiatric disorders [12]. The brain imaging-derived phenotypes (IDPs) may play a mediating role between inactivity and MDD. Hence, an analysis of this aspect will help to prevent the risks and understand the mechanism between inactivity and MDD.

In this study, we aimed to examine the bidirectional causal effects between MDD and different types of leisure/social activity, physical activity, or “none of the above” (leisure/social inactivity or physical inactivity). We also assessed the mediating effect of obesity-related measures and IDPs on the association between different types of activities or inactivity and MDD.

## Methods

**Fig. 1:**
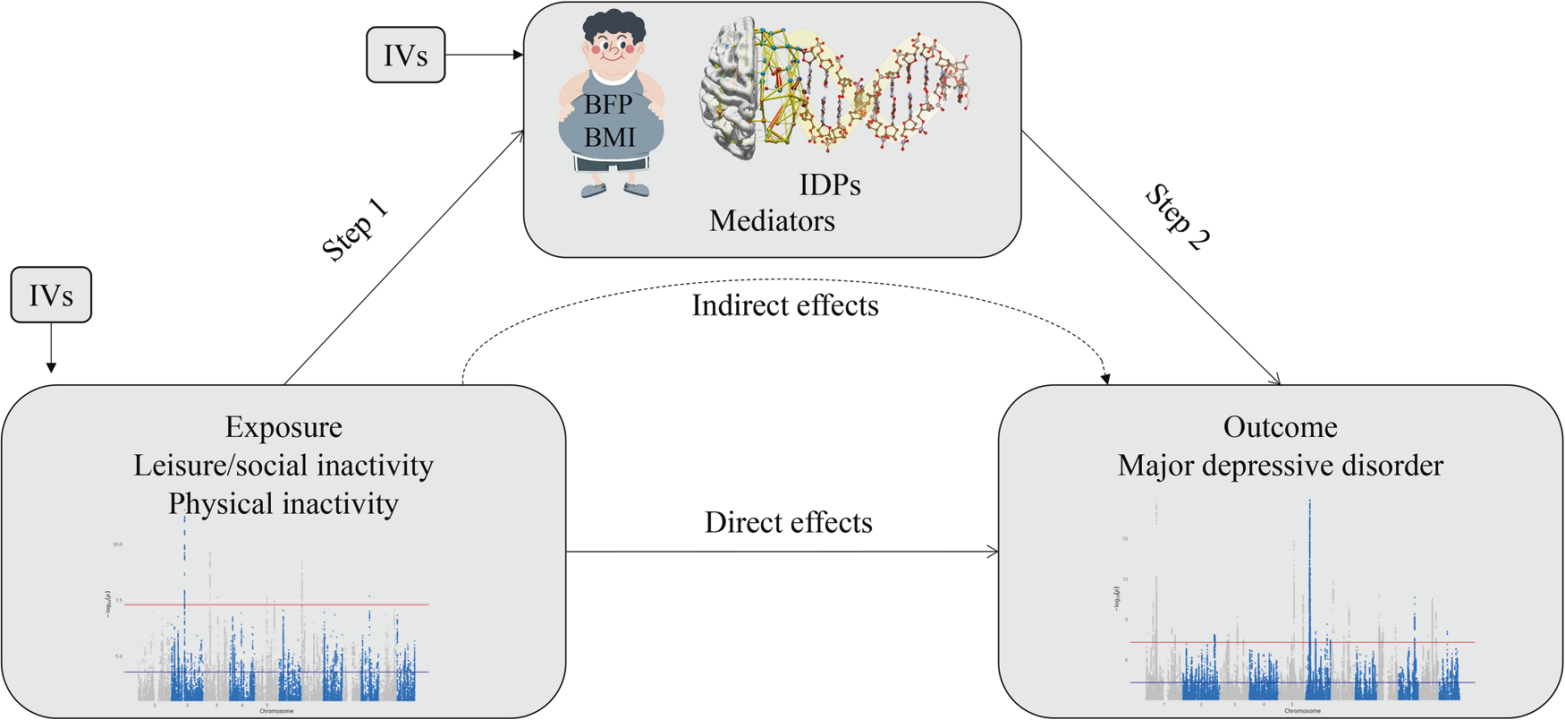
Two-step MR analysis framework for inactivity. BFP body fat percentage, BMI body mass index, IDPs brain imaging-derived phenotypes, IVs instrumental variables, SNP single nucleotide polymorphism.

This two-sample MR was based on data from publicly available GWAS summary statistics, and ethical approval was obtained from the original studies. Details of the data sources used in this study are summarized in Supplementary Table S1. We extracted leading SNPs associated with social/physical activities and MDD as genetic IVs to perform bidirectional two-sample MR (Supplementary Fig. S1). Moreover, two-step MR analyses were used to assess whether obesity metrics and IDPs play a role as mediators between inactivity and MDD (Fig. 1). In addition, to minimize racial mismatches, the current analyses were restricted to participants of European descent.

To filter eligible genetic instruments, we performed a series of quality controls. First, we included SNPs that were strongly associated with exposure factors with *P* < 5 × 10^−8^. Second, we conducted clumping with *R*^2^ < 0.001 and a window size of 10,000 kb. Third, proxy SNPs (*R*^2^ > 0.8) replaced the SNPs of exposure when the targeted SNPs were not found in the outcome datasets. Fourth, the minor allele frequency of SNPs found in the outcome GWAS was set to a default of 0.3. Finally, we harmonized the exposure and outcome to eliminate palindromic SNPs and ensure that the effect alleles belonged to the same allele [19]. We also investigated each IV in the PhenoScanner GWAS database [20] and removed the SNPs associated with plausible confounders (such as alcohol consumption and smoking) [21]. The application of less than 10 independent SNPs as IVs may decrease statistical efficiency in MR analysis [22]. Therefore, we use a relaxed and previously used instrument threshold (*P* < 1 × 10^−5^) [23] to ensure the number of SNPs, when traits lacked sufficient SNPs (≤10).

Furthermore, to quantify the strength of IVs, we calculated the *F*-statistics of all genetic instruments using the genetic variants (*R*^2^), sample size (*N*), and the number of instruments (*k*) using the formula *F* = *R*^2^(*N-k-1*)/*k*(*1-R*^2^) [24]. When the F-statistic was less than 10, the genetic variant was considered weak, which may bias the results [25].

We utilized publicly available GWAS summary-level data associated with leisure/social activities, including sport clubs or gyms, pubs or social clubs, religious groups, adult education classes, other group activities, and “none of the above” (leisure/social inactivity), from the Medical Research Council Integrative Epidemiology Unit (MRC-IEU) UK biobank (Supplementary Table S1) entailing 461,369 participants. An individual who selected “none of the above” on the leisure/social activities questionnaire means that he or she participated in leisure/social activities less than once a week in the last year, which we call “leisure/social inactivity”. Genetic variants associated with types of physical activity in the last 4 weeks from the MRC-IEU were also used to identify the instruments. The questionnaire on types of physical activities included walking for pleasure (not as a means of transport), strenuous sports (sports that make you sweat or breathe hard), light “do-it-yourself” (DIY; such as pruning and watering the lawn), heavy DIY (such as weeding, lawn mowing, carpentry, and digging), other exercises (such as swimming, cycling, keeping fit, bowling), and “none of the above” (physical inactivity), consisting of 460,376 individuals (Supplementary Table S1). Choosing “none of the above” in this questionnaire means refusing any form of physical activity for the past four weeks, which we call “physical inactivity”. The summary information of IVs was showed in the Supplementary Tables S2–S13.

Summary statistics for MDD were obtained from a meta-analysis of GWAS involving 170,756 cases and 329,443 controls of European descent, which included participants from the Psychiatric Genomics Consortium (PGC) and UK Biobank (excluding 23andme) [26]. When the genetic variables associated with MDD were used as instruments, a total of 49 SNPs were identified to be significantly associated with MDD (*P* < 5 × 10^−8^), and 47 SNPs satisfied the standard for IVs (Supplementary Table S14). For avoid overlapping sample, when MDD was outcome, we used the other GWAS summary data for MDD removed UK Biobank and 23andMe samples to conduct a series of subgroup analyses, which included subsample of 143,265 (45,591 cases and 97,674 controls) [27]. The IVs associated with the potential confounders are listed in Supplementary Table S15.

We used publicly available GWAS summary statistics of potential mediators (Supplementary Table S1), including body fat percentage (BFP) in 454,633 individuals, body mass index (BMI) in 461,460 samples, hip circumference (HP) in 462,117 individuals, waist circumference (WC) in 462,166 participants from MRC-IEU [28], and waist-to-hip ratio (WHR) in 212,244 individuals from the Genetic Investigation of ANthropometric Traits (GIANT) [29]. For brain imaging phenotypes, we used the summary data of 3144 functional and structural brain IDPs from UK Biobank (discovery dataset 8428 subjects) [30], which covered the whole brain and including multimodal information on white matter connections, functional connectivity, grey matter volume, area and thickness. The summary statistics of IDP were available from the Oxford Brain Imaging Genetics (BIG) web browser (http://big.stats.ox.ac.uk/) [30].

### Statistical analyses

All analyses were conducted using the *TwoSampleMR* R package [31]. Our primary MR method was inverse-variance weighted (IVW) regression [32], which is currently common and easy 2-sample MR method and often is used as major analysis method in many studies [18, 33, 34]. In addition, we also adopted complementary methods, including the MR-Egger regression, weighted median, simple mode, and weighted mode, to estimate the causal effects of exposure on outcomes [35–37]. A detailed description of these methods is attached in the Supplementary methods.

Next, we performed a series of sensitivity analyses to assess the robustness and pleiotropy of causal estimates. We used a random-effect modal IVW MR analysis when Cochran’s Q statistic suggested the heterogeneity of different genetic variants [38]. We also performed MR-egger intercept test for directional pleiotropy, which would be proved when the intercept term deviated from zero (*P* < 0.05) [39, 40]. A leave-one-out IVW regression was used to test the robustness of the results. In addition, we utilized the Pleiotropy Residual Sum and Outlier (MR-PRESSO) analysis [41] to detect the outlier SNPs reflecting likely pleiotropy with a *P* value of 0.05, and correct for horizontal pleiotropy by removing the outlier. Associations with *P* values below 0.004 (0.05/12) were deemed statistically significant evidence of a causal association. *P* value between 0.004 and 0.05 was regarded as evidence of potential causal association [23]. Additionally, MR estimates could be subject to different types of biases due to the overlap between the exposure and outcome samples [42]. Thus, we used the *MRlap* R package to account and correct biases due to the overlapping samples (see supplementary methods) [43].

For all the significant association MR results of different activities on MDD, we conducted two-step MR analyses to detect the potential mediating effects of obesity metrics. When the exposures influenced the mediator, which in turn influenced MDD risk, we used the “product of coefficients” [44]. In addition, for inactivity on MDD, 3144 functional and structural IDPs were regarded as mediators to conduct two-step MR (Fig. 1). We extracted IDPs where both steps MR were significant (*P* < 0.05) and overlapping as mediators and examined the significance of the heritability of these IDPs.

In the current analysis, we expressed our MR results as odds ratios (ORs) accompanied by their respective 95% confidence intervals (CIs), which represent the outcome risk associated with unit changes in exposure. Since all exposure variables in this study are binary, we interpreted the final effect estimates as ORs on the outcome risk per log-OR change in exposure [45].

## Results

### Causal effects of different activities on MDD

**Fig. 2:**
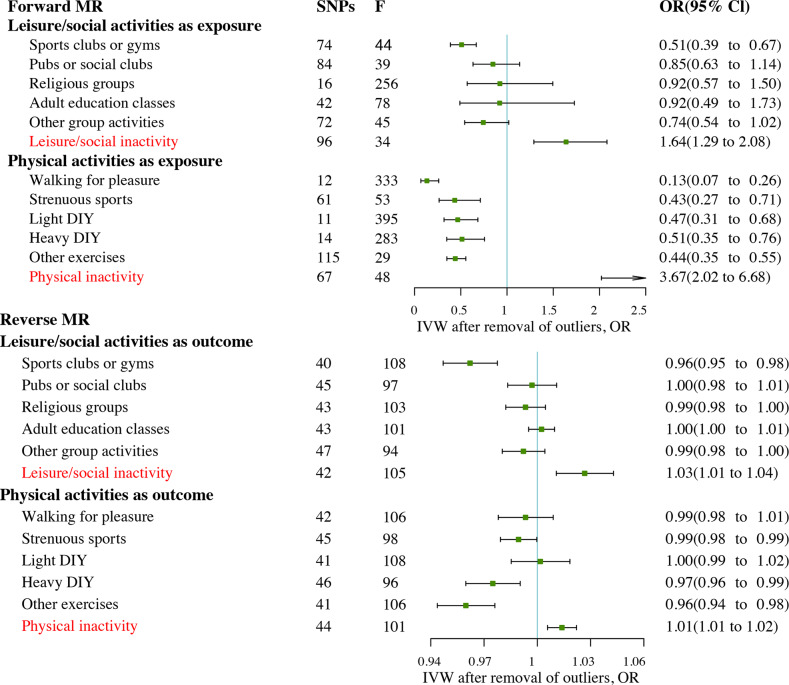
Bidirectional MR estimates between different activities and MDD with outliers removed. CI confidence interval, IVW inverse variance weighted method, MDD major depressive disorder, OR odds ratio, SNP single nucleotide polymorphism.

**Fig. 3:**
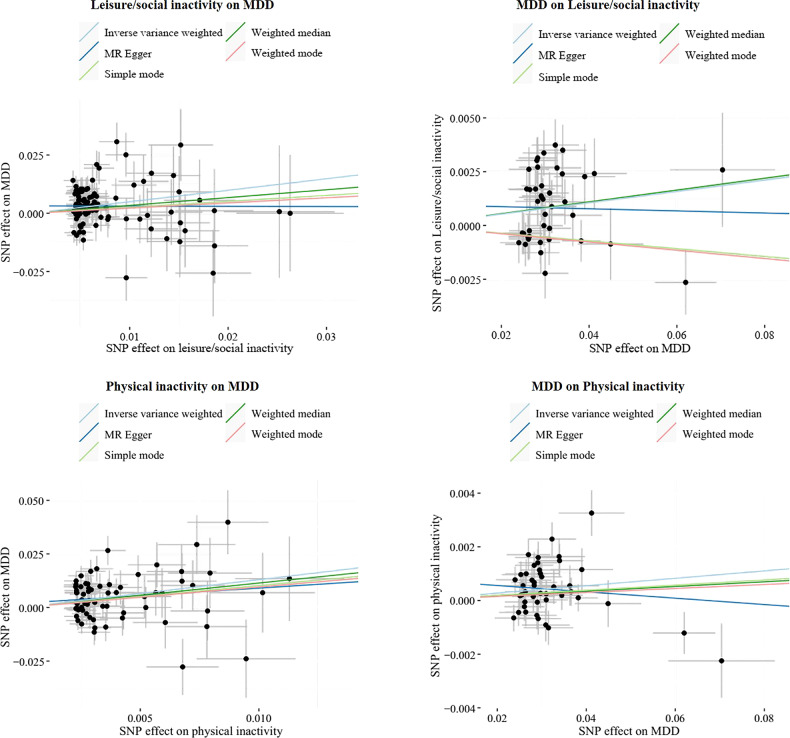
The scatter plot of SNP effects between inactivity and MDD after removing outliers. The slope of each line was corresponding to the estimated MR effect per method. The data are expressed as raw *β* values with 95% confidence interval. MDD, major depressive disorder.

After removing outliers, MR analysis showed a protective causal relationship between attending sports clubs or gyms. Per log-OR increment in attending sports clubs or gyms was associated with 49% lower risk of MDD (IVW OR = 0.51; 95% CI, 0.39 to 0.67; *P* = 1.05 × 10^−6^) and MDD (Fig. 2). However, the OR for MDD of genetically predicted per log-OR increase in leisure/social inactivity was 1.64 (95% CI, 1.29 to 2.08; *P* = 5.14 × 10^−5^) (Fig. 3, Supplementary Table S16 and Figures S2, S3). There was no statistically significant difference in the rest of the leisure/social activities.

After removal of outliers (Fig. 2, Supplementary Table S17 and Figures S2, S3), MR analysis identified protection from physical activities of walking for pleasure (IVW OR = 0.13; 95% CI, 0.07 to 0.26; *P* = 4.25 × 10^−9^), strenuous sports (IVW OR = 0.43; 95% CI, 0.27 to 0.71; *P* = 9.16 × 10^−4^), light DIY (IVW OR = 0.47; 95% CI, 0.32 to 0.68; *P* = 9.65 × 10^−5^), Heavy DIY (IVW OR = 0.51; 95% CI, 0.35 to 0.76; *P* = 7.46 × 10^−3^), or other exercises (IVW OR = 0.44; 95% CI, 0.35 to 0.55; *P* = 3.30 × 10^−12^) on MDD. However, per log-OR increase in physical inactivity was associated with a 3.67-fold increase in the risk of MDD (IVW OR = 3.67; 95% CI, 2.02 to 6.68; *P* = 1.99 × 10^−5^; Fig. 3). All MR-Egger intercept tests indicated no horizontal pleiotropy. SNPs exclusion demonstrated the stability of the results (Supplementary Fig. S4). The *F*-statistics found no weak instrument bias. Apart from walking for pleasure and light DIY, the results of the subgroup analyses without overlapping samples remained significant (for detail see Supplementary Table S18). The results of *MRlap* showed the differences between observed and corrected effects were not significant (Supplementary Table S19).

### Causal effects of MDD on different activities

In the other direction, genetic liabilities for MDD were used as exposures. After excluding outliers, the MR results showed a protective causal relationship between MDD and sport clubs or gyms (IVW OR = 0.96; 95% CI, 0.95 to 0.98; *P* = 1.82 × 10^−6^), but the OR of leisure/social inactivity of genetically predicted per log-OR increase in MDD was 1.03 (95% CI, 1.01 to 1.04; *P* = 9.89 × 10^−4^; Figs. 2, 3, Supplementary Table S20 and Figs. S5, S6). Results were not statistically significant for the effect of MDD on the rest of leisure/social activities.

With genetic variants for different types of physical activities as outcomes, the causal estimates from MDD to heavy DIY (IVW OR = 0.97; 95% CI, 0.96 to 0.99; *P* = 1.54 × 10^−3^) or other exercises (IVW OR = 0.96; 95% CI, 0.94 to 0.98; *P* = 1.88 × 10^−6^) were significant, after removing outliers. MDD was associated with lower likelihood of attending strenuous sports (IVW OR = 0.99; 95% CI, 9.79 × 10^−1^ to 9.99 × 10^−1^; *P* = 4.41 × 10^−2^). In addition, MR also revealed a causal relationship between MDD and physical inactivity (IVW OR = 1.01.; 95% CI, 1.01 to 1.02; *P* = 7.96 × 10^−4^) (Fig. 3). The MR results showed no significant effects of MDD on light DIY or walking for pleasure, even after removing outliers (Supplementary Table S21). The *F*-statistics found no weak instrument bias. Furthermore, all the MR-Egger intercept tests indicated no horizontal pleiotropy. The analyses excluding each SNP indicated that no single SNP drove these results (Supplementary Fig. S7).

### Mediation analysis

**Fig. 4:**
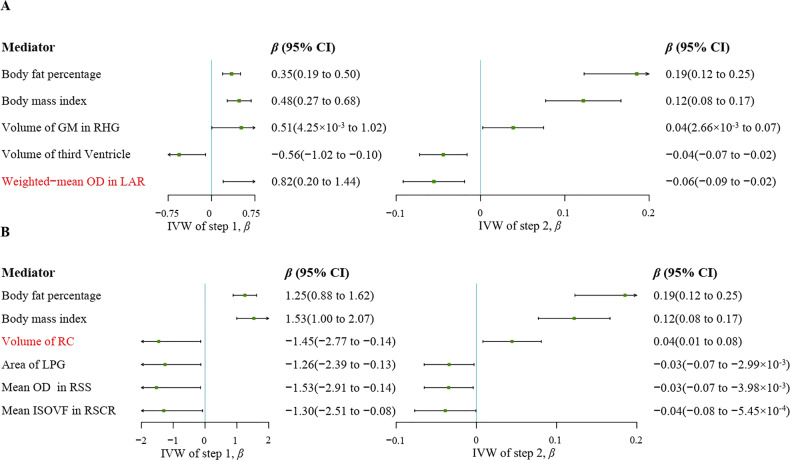
Summary two-step MR estimates derived from the IVW methods for the effect of inactivity on MDD. ‘A’ indicates two-step MR results of leisure/social inactivity on MDD. ‘B’ indicates two-step MR results of physical inactivity on MDD. BFP, body fat percentage; BMI, body mass index; CI, confidence interval; IVW, inverse variance weighted method; MDD, major depressive disorder; MR, Mendelian Randomization; Volume of GM in RHG, volume of grey matter in right heschl’s gyrus; Weighted-mean OD in LAR, weighted-mean orientation dispersion index in tract left acoustic radiation; Volume of RC, volume of right caudate; Area of LPG, area of precentral gyrus in the left hemisphere; Mean OD in RSS, mean orientation dispersion index in right sagittal stratum; Mean ISOVF in RSCR, mean isotropic or free water volume fraction in right superior corona radiata.

We conducted a two-step MR analysis to investigate a causal relationship between the different activity types and MDD through obesity-related metrics. First, instruments for different types of activities were used to estimate the causal relationship between exposure and potential mediators. Across all types of activities and the mediators, we identified significant causal effects (Supplementary Tables S22–S26). Second, genetic instruments for obesity-related measurements were used to assess the causal effect of mediators on MDD risk. We found a relationship between BFP, BMI, HC, or WC and MDD (Supplementary Table S27). Given that obesity-related measurements are related to each other, we conducted a multivariable MR analysis to estimate the direct effect of each on MDD. Among the potential mediators, only BFP (OR = 1.34; 95% CI, 1.07 to 1.67; *P* = 0.01) and BMI (OR = 1.23; 95% CI, 1.02 to 1.50; *P* = 0.03) reached significance (Supplementary Table S28). Finally, we estimated the indirect effect of the types of activity on MDD via BFP or BMI (Supplementary Table S29). For leisure/social inactivity, we found the mediation effect of BFP was 0.06 (95% CI, 0.02 to 0.12) with a mediated proportion of 12.99% (Table 1 and Fig. 4) and the mediation effect of BMI was 0.06 (95% CI, 0.02 to 0.11) with a mediated proportion of 11.80%. The mediation effects of BFP and BMI were 0.23 (95% CI, 0.11 to 0.40) and 0.19 (95% CI, 0.08 to 0.34) between physical inactivity and MDD, and the effect proportions were 17.82% and 14.38% respectively.

**Table 1:** The mediation effect for types of physical activity on MDD via BFP or BMI.

Mediator	Total effect *β* (95% CI)	Direct effect A *β* (95% CI)	Direct effect B *β* (95% CI)	Mediation effect *β* (95% CI)	Types of Mediation effect	Effect proportion (%)
Leisure/social inactivity as exposure						
Body fat percentage	0.49(0.25 to 0.73)	0.35(0.19 to 0.50)	0.19(0.12 to 0.25)	0.06(0.02 to 0.12)	Partial mediation	12.99%
Body mass index	0.49(0.25 to 0.73)	0.48(0.27 to 0.68)	0.12(0.08 to 0.17)	0.06(0.02 to 0.11)	Partial mediation	11.80%
Volume of GM in RHG	0.49(0.25 to 0.73)	0.51(4.25 × 10^−3^ to 1.02)	0.04(2.66 × 10^−3^ to 0.07)	0.02(1.13 × 10^−5^ to 0.08)	Partial mediation	4.03%
Volume of third Ventricle	0.49(0.25 to 0.73)	−0.56(−1.02 to −0.10)	−0.04(−0.07 to −0.02)	0.02(1.63 × 10^−3^ to 0.07)	Partial mediation	5.01%
Weighted-mean OD in LAR	0.49(0.25 to 0.73)	0.82(0.20 to 1.44)	−0.06(−0.09 to −0.02)	−0.05(−0.13 to −3.81 × 10^−3^)	Masking effect	8.43%
Physical inactivity as exposure						
Body fat percentage	1.30(0.70 to 1.90)	1.25(0.88 to 1.62)	0.19(0.12 to 0.25)	0.23(0.11 to 0.40)	Partial mediation	17.82%
Body mass index	1.30(0.70 to 1.90)	1.53(1.00 to 2.07)	0.12(0.08 to 0.17)	0.19(0.08 to 0.34)	Partial mediation	14.38%
Volume of RC	1.30(0.70 to 1.90)	−1.45(−2.77 to −0.14)	0.04(0.01 to 0.08)	−0.06(−0.22 to −1.12 × 10^−3^)	Masking effect	4.74%
Area of LPG	1.30(0.70 to 1.90)	−1.26(−2.39 to −0.13)	−0.03(−0.07 to −2.99 × 10^−3^)	0.04(3.89 × 10^−4^ to 0.16)	Partial mediation	3.29%
Mean OD in RSS	1.30(0.70 to 1.90)	−1.53(−2.91 to −0.14)	−0.03(−0.07 to −3.98 × 10^−3^)	0.05(5.72 × 10^−4^ to 0.19)	Partial mediation	4.05%
Mean ISOVF in RSCR	1.30(0.70 to 1.90)	−1.30(−2.51 to −0.08)	−0.04(−0.08 to −5.45 × 10^−4^)	0.05(4.35 × 10^−5^ to 0.19)	Partial mediation	3.86%

Note: ‘direct effect A’ indicates the effect of inactivity on mediators, ‘direct effect B’ indicates the effect of mediators on MDD; *BFP* body fat percentage, *BMI* body mass index, *MDD* major depressive disorder, *Volume of GM in RHG* volume of grey matter in right heschl’s gyrus, *Weighted-mean OD in LAR* weighted-mean orientation dispersion index in tract left acoustic radiation, *Volume of RC* volume of right caudate, *Area of LPG* area of precentral gyrus in the left hemisphere, *Mean OD in RSS* mean orientation dispersion index in right sagittal stratum, *Mean ISOVF in RSCR* mean isotropic or free water volume fraction in right superior corona radiata.

In addition, 3144 IDPs were used as mediators to examine the mediating pathway between inactivity and MDD. First, instruments for inactivity were used to estimate the causal relationship between inactivity and brain imaging phenotypes. Across all 3144 IDPs, we identified significant causal effects from leisure/social inactivity or physical inactivity to IDPs (Supplementary Tables S30, S31). Second, genetic instruments for IDPs were used to assess the causal effect of mediators on MDD risk (Supplementary Table S32). And then, the significant and overlapping IDPs in both steps MR were extracted and examined their heritability (Supplementary Table S33). Finally, only seven IDPs met the criteria (Fig. 4). The mediation analyses showed the relationship of leisure/social inactivity on MDD was partly mediated by volume of grey matter in right heschl’s gyrus (4.03%) and volume of third ventricle (5.01%). Due to the indirect and direct effects of weighted-mean orientation dispersion index (OD) of left acoustic radiation showing opposite signs, we concluded it as masking effect [46], and the effect proportion was 8.43%. For the physical inactivity on MDD, we found the partial mediation effect of the surface area in left precentral gyrus (3.29%), mean OD in right sagittal stratum (4.05%), and mean isotropic or free water volume fraction (ISOVF) in right superior corona radiata (3.86%). The volume of right caudate has a masking effect with a effect proportion of 4.74% (Table 1).

## Discussion

In this MR study, significant potential causal effects between different types of leisure/social or physical activities and MDD were identified. We detected protective effects from attending sports clubs or gyms, strenuous sports, heavy DIY and other exercises. In contrast, MR results showed a significant potential causal relationship between MDD and reduced leisure/social or physical activities, such as sport clubs or gyms attendance, strenuous sports, heavy DIY, and other exercises. Unlike previous study [18], we focused on types of activity, particularly inactivity. We observed bidirectional causal effect between recent leisure/social or physical inactivity and MDD. In addition, we focused on the risk mechanisms of inactivity and found that the effects of activity or inactivity on MDD are partially mediated by BFP and BMI. Some IDPs played a mediating or masking role between inactivity and MDD. Our results proved robust after a series of sensitivity analyses and pleiotropy assessment.

Our study showed a significant protective causal effect from attending sports clubs or gyms to MDD. Specifically, Kleppang et al. suggested attending sports club was associated significantly with lower odds for symptoms of MDD, which means social activity might play a vital role in addition to physical activity [47]. Attending leisure/social activities also often entail a certain amount of physical activity, and our work showed that physical activity is protective against MDD. However, a prior MR study suggested that the causal relationship between self-reported activity and MDD is not significant [6], perhaps owing to the differences in the strength of genetic instruments or self-report measures. In the current study, majority MR results from physical activity types to MDD were significant, which provide that physical activities are effective in preventing MDD [5]. This is important for public health. A previous study also showed that approximately 12% of MDD cases could be prevented through an hour of physical activity every week [48].

We also aimed to elucidate the direction of the estimated causal effects between physical/social activities and MDD. Through bidirectional MR, we found that patients with MDD may lead less likely to attend sport clubs or gyms, strenuous sports, heavy DIY, and other exercises, although this effect is not great. These are consistent with the symptoms of MDD, such as loss of energy and interest in previously enjoyed activities [11]. Another study also showed that depressive symptoms were associated with sedentariness and reduced physical activity [49]. Interestingly, sport clubs or gyms, strenuous sports, heavy DIY, and other exercises all showed evidence of a bidirectional causality; that is, attending these activities reduced the risk of MDD, but MDD also reduced the occurrence of these events.

To our knowledge, this study is the first to investigate the potential bidirectional causal relationship between social/physical inactivity and MDD using the MR method. The protective effects of social activities and larger social networks in MDD have been demonstrated [4, 18]. As an extension, we explored the causal relationship between not participating in leisure/social activities and MDD. In addition, a previous study has shown that people who participated less in sport clubs or physical/social activities exhibited higher odds of symptoms of MDD [50]. In this case, the physical aspect could trump the social one. Our results also suggest that physical inactivity may increase the risk of MDD. Inactivity in the last 4 weeks often indicated a lack of exercise or sedentary behavior. Sedentary behavior is slightly different from lack of exercise, though they have both been significantly associated with MDD and anxiety [51]. One study [52] indicated that mentally passive sedentary behaviors (such as watching TV or sitting around) increased the risk of MDD, whereas mentally active sedentary behaviors (such as reading or driving) do not increase the risk of MDD [52]. Conversely, MDD increased the risk of social/physical inactivity, which might lead to a sedentary lifestyle [49, 53]. There seems to be a vicious circle in which inactivity increases the risk of MDD, and MDD itself may be a reason for avoidance of social/physical activities. Physiological links between a lack of activity and MDD include hypothalamic-pituitary-adrenal (HPA) axis and sympathetic nervous system hyperactivity, increased inflammatory markers, and lower pain thresholds [53]. Further investigation is needed to determine how these underlying mechanisms may contribute to the potential bidirectional causality.

Two-step MR for mediation analysis showed that the protective effect of leisure/social or physical activities was partially mediated by decreased BFP and BMI, and that inactivity increased the risk of MDD in part by raising BFP and BMI. A recent MR study reported that moderate-vigorous physical activities lower the BMI, and longer sedentary time increases the BMI [54], which is consistent with the first-step MR. MR studies showed causal risk factors for BMI or BFP in MDD [55, 56], which agrees with our findings. Interestingly, when the IDPs were regarded as mediators, we found that leisure/social inactivity increased the weighted-mean OD in tract left acoustic radiation, which in turn leads to a reduction of risk of MDD. In other words, the presence of increased weighted-mean OD in tract left acoustic radiation seems to mitigate the total effects of leisure/social inactivity on MDD. Similarly, volume of right caudate played a masking role between physical inactivity and MDD, which means the decreased volume of the right caudate may attenuate the effect of physical inactivity on MDD [46]. Besides, studies also showed that exercise could reshape the brain structure of MDD patients and maintain the integrity of hippocampal and white matter, which helped promote behavioral adaption changes [57].

Our study had some limitations. First, although strongly associated SNPs were selected, they cannot be considered exact proxies for exposure [58]. Additionally, we also could not determine whether physical activity in the past 4 weeks is representative of long-term lifestyle habits of individuals. The MDD metric used in our study measures long-term effects, and further research could differentiate between first-episode depression and relapse to better showcase the risk associated with inactivity. Second, our study relied primarily on self-reporting, which may not be able to exclude emotional states and cognitive biases that can also influence mental health. Although this does not diminish the usefulness of self-reported measures, the accelerometer-based activity study by Choi et al. [6] also suggests that we should consider objectively measured actigraphy data in future studies to consider inactivity in a more accurate manner. Third, UK Biobank summary data was used in this study only included European ancestry population, hence, whether the results can be generalized to other populations remains to be verified. Moreover, we were not able to determine whether gender would bias the results due to the summary data lacking sufficient information. Fourth, in order to obtain sufficient SNPs and ensure the accuracy of the results, we sometimes had to relax the threshold. It is difficult to completely eliminate potential pleiotropy due to lack of knowledge of potential confounders and inability to get individual level data. [59]. Nevertheless, we inferred robust causal effects by outlier removal using MR-PRESSO and a series of sensitivity analyses. Fifth, there were likely overlapping samples in the exposure and outcome studies, which could have resulted in weak instrument bias. To prevent this, we used strong instruments (*F* statistic much greater than 10) [60], subgroup analysis without overlapping samples, and *MRlap* approach. Finally, we used binary variables in the study, which led to our inability to determine more specific risk effect parameters (such as time and frequency of activity). Additionally, although some MR results were significant when MDD was used as an exposure, the effect sizes were small and more studies are needed to validate them.

## Conclusions

We found potential bidirectional causal relationships between sports clubs or gyms, strenuous sports, heavy DIY, other exercises and MDD. Social or physical activity reduced the risk of MDD, but MDD was a cause of reduced social and physical activity. This bidirectional causal effect between social or physical inactivity and MDD may be evidence of a vicious cycle that may account for the steady increase in the burden of MDD. These results provided support for the development of novel prevention and intervention strategies for MDD. In addition, the mediating effect of obesity-related measures or IDPs provided more information to understand the mechanism of inactivity on MDD.

## Supplementary information

Supplementary material

Supplementary table

## Data Availability

All GWAS summary statistics analyzed in this study are publicly available for download by qualified researchers.

## Code availability

All the analyses used in this study were performed R packages *TwoSampleMR* (version 0.5.6), MR-PRESSO (version 1.0) and MRlap (version 0.0.3.0). The code is available at https://mrcieu.github.io/TwoSampleMR/ and https://github.com/n-mounier/MRlap.
