# Supplementary material for: Dissecting the causal association between social or physical inactivity and depression: a bidirectional two-sample Mendelian Randomization study
Source: Transl Psychiatry. 2023 Jun 8;13:194. doi: 10.1038/s41398-023-02492-5 (PMC10250407; doi:10.1038/s41398-023-02492-5)
Supplement: Supplementary file 1 — Supplementary material [file 41398_2023_2492_MOESM1_ESM.doc]

*Supplementary*

**Dissecting the causal association between social or physical inactivity and depression: A bidirectional two-sample Mendelian Randomization study**

Guorui Zhao ^1,2,3†^, BMed, Zhe Lu ^1,2,3†^, MMed, Yaoyao Sun ^1,2,3^, Ph.D, Zhewei Kang ^1,2,3^, MMed, Xiaoyang Feng ^1,2,3^, MMed, Yundan Liao ^1,2,3^, BMed, Junyuan Sun ^1,2,3^, MMed, Yuyanan Zhang ^1,2,3*^, Ph.D, Yu Huang ^4*^, Ph.D,Weihua Yue ^1,2,3,5,6*^ Ph.D.

^1^ Peking University Sixth Hospital, Peking University Institute of Mental Health, Beijing, 100191, China

^2^ National Clinical Research Center for Mental Disorders (Peking University Sixth Hospital), Beijing, 100191, China

^3^ NHC Key Laboratory of Mental Health (Peking University), Beijing, 100191, China

^4^ National Engineering Research Center for Software Engineering, Peking University, Beijing 100871, China

^5^ PKU-IDG/McGovern Institute for Brain Research, Peking University, Beijing, 100871, China

^6^ Chinese Institute for Brain Research, Beijing, 102206, China

* Correspondence to:

Prof. Weihua Yue, Institute of Mental Health, Peking University Sixth Hospital, No. 51 Hua Yuan Bei Road, Beijing 100191, P.R. China. Tel: +86-10-8280-5307. E-mail: [dryue@bjmu.edu.cn](mailto:dryue@bjmu.edu.cn) or [zhang_yyn@bjmu.edu.cn](mailto:zhang_yyn@bjmu.edu.cn).

Prof. Yu Huang, National Engineering Research Center for Software Engineering, Peking University, Beijing, 100871, P.R. China. E-mail: [hy@pku.edu.cn](mailto:hy@pku.edu.cn).

^†^ These authors contributed equally to this work.

**Contents**

[Supplementary methods 3](#__RefHeading___Toc135785784)

[Supplementary figures list 4](#__RefHeading___Toc135785785)

[Figure S1 Mendelian randomization (MR) model and two-step MR analysis framework. 4](#__RefHeading___Toc135785786)

[Figure S2 The scatter plot of SNP effects on different activities versus MDD after removing outliers 5](#__RefHeading___Toc135785787)

[Figure S3 The forest plots of the association between genetic predicted different activities on MDD after removing outliers 6](#__RefHeading___Toc135785788)

[Figure S4 Leave-one-out analyses for SNPs associated with different activities on MDD after removing outliers 7](#__RefHeading___Toc135785789)

[Figure S5 The scatter plot of SNP effects on MDD versus different activities after removing outliers 8](#__RefHeading___Toc135785790)

[Figure S6 The forest plots of the association between genetic predicted MDD on different activities after removing outliers 9](#__RefHeading___Toc135785791)

[Figure S7 Leave-one-out analyses for SNPs associated with MDD on different activities after removing outliers 10](#__RefHeading___Toc135785792)

# Supplementary methods

IVW method combines the Wald ratio estimates for each SNP into one causal estimate for each risk factor and provides a more robust causal estimate in the absence of pleiotropy [1, 2]. MR-Egger relaxes the assumption of “no horizontal pleiotropy”, and allowing a non-zero intercept. It returns a pleiotropy-robust causal effect even if the “no horizontal pleiotropy assumption” is violated for all SNPs [3]. The weighted median method takes the median effect of all available SNPs, which only needs half of the SNPs to be valid instruments [4]. Furthermore, although weighted median and MR-Egger methods are relatively robust for horizontal pleiotropy, they come at the cost of reduced statistical power [5]. Finally, the Mode-based method cluster the SNPs into subsets resting on the resemblance of causal effects, and estimates the causal effect based on the cluster with the largest number of SNPs [6].

*MRlap* is an R-package to perform two-sample Mendelian Randomization (MR) analyses using (potentially) overlapping samples, relying only on GWAS summary statistics (https://github.com/n-mounier/MRlap). MR estimates can be subject to different types of biases due to the overlap between the exposure and outcome samples, the use of weak instruments and winner’s curse. This approach simultaneously accounts and corrects for all these biases, using cross-trait LD-score regression (LDSC) to approximate the overlap. Estimating the corrected effect using our approach can be performed as a sensitivity analysis: if the corrected effect do not significantly differ from the observed effect, then IVW-MR estimate can be safely used. However, when there is a significant difference, corrected effects should be preferred as they should be less biased, independently of the sample overlap [7].

# Supplementary figures list


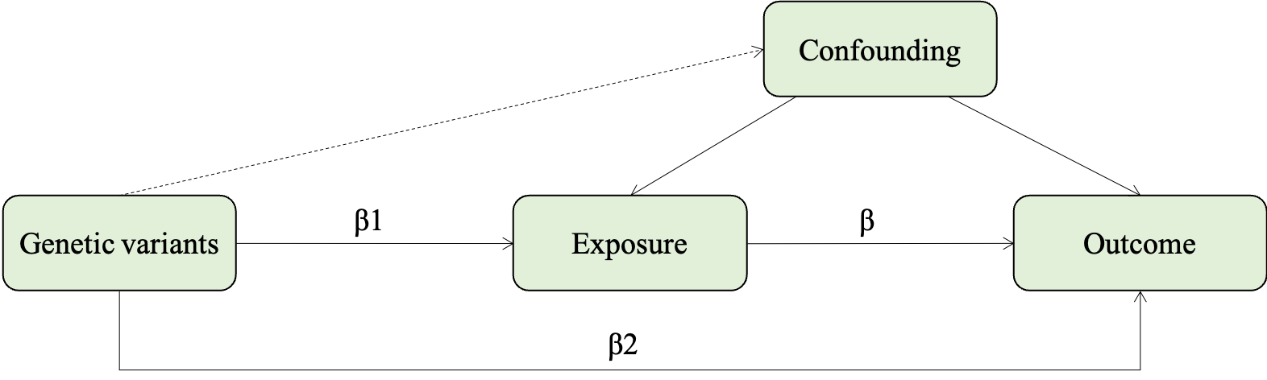


Figure S1 Mendelian randomization (MR) model and two-step MR analysis framework. A) Mendelian randomization illustration. There are three principal assumptions in MR design, namely genetic instrumental variables should [1] have a strong link to the exposure, [2] be not associated with confounders linked to the chosen exposure and outcome, and 3) influence the outcome only via exposure. β1and β2 denote to the gene–exposure and gene–outcome association, respectively; β represents the causal association between exposure and outcome, where β= β2/β1.


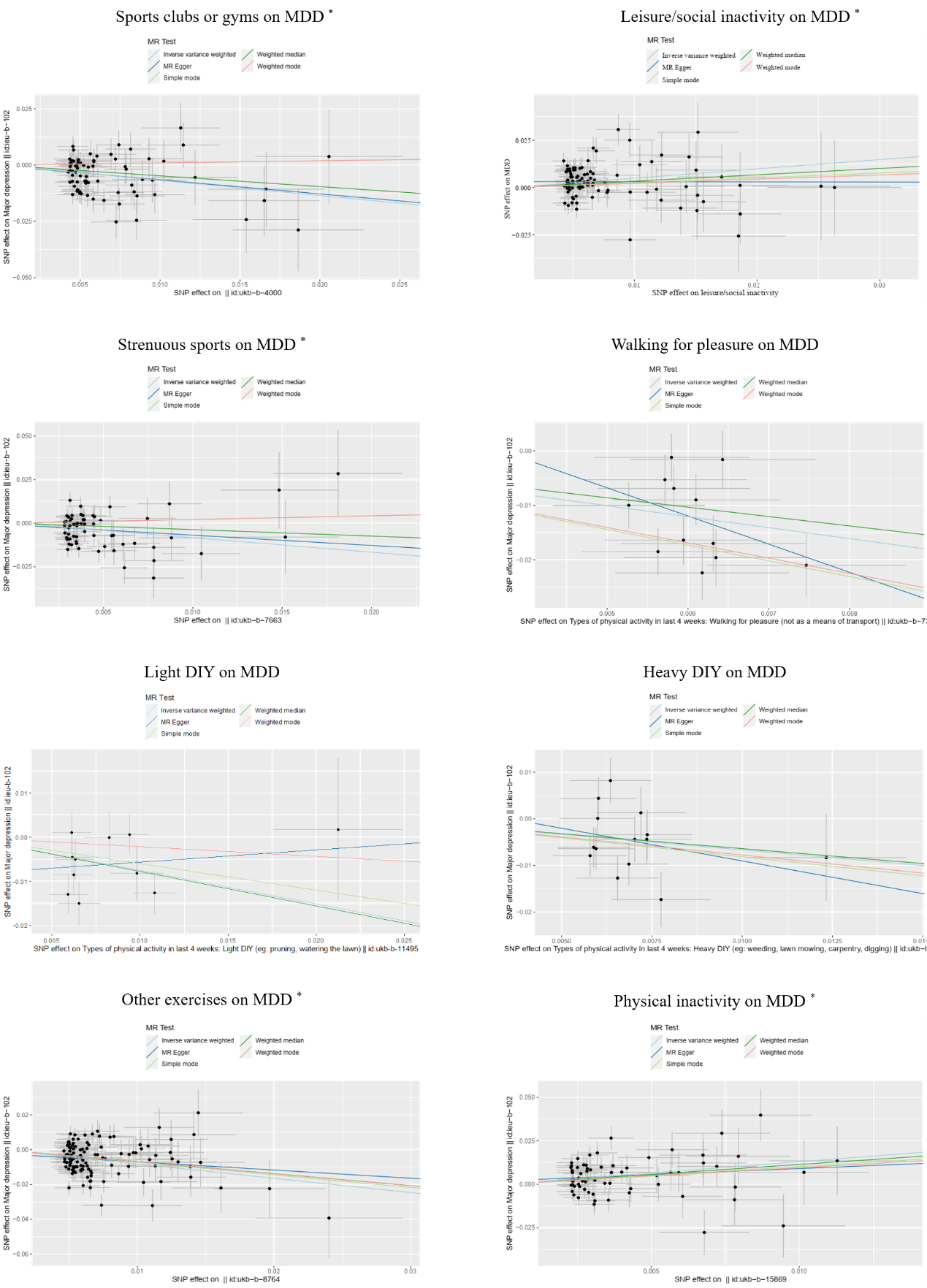


### Figure S2 The scatter plot of SNP effects on different activities versus MDD after removing outliers

Note: The slope of each line was corresponding to the estimated MR effect per method. The data are expressed as raw β values with 95% confidence interval. ^*^ Using a relaxed instrument threshold (*P*< 1×10^-5^); MDD, major depressive disorder.


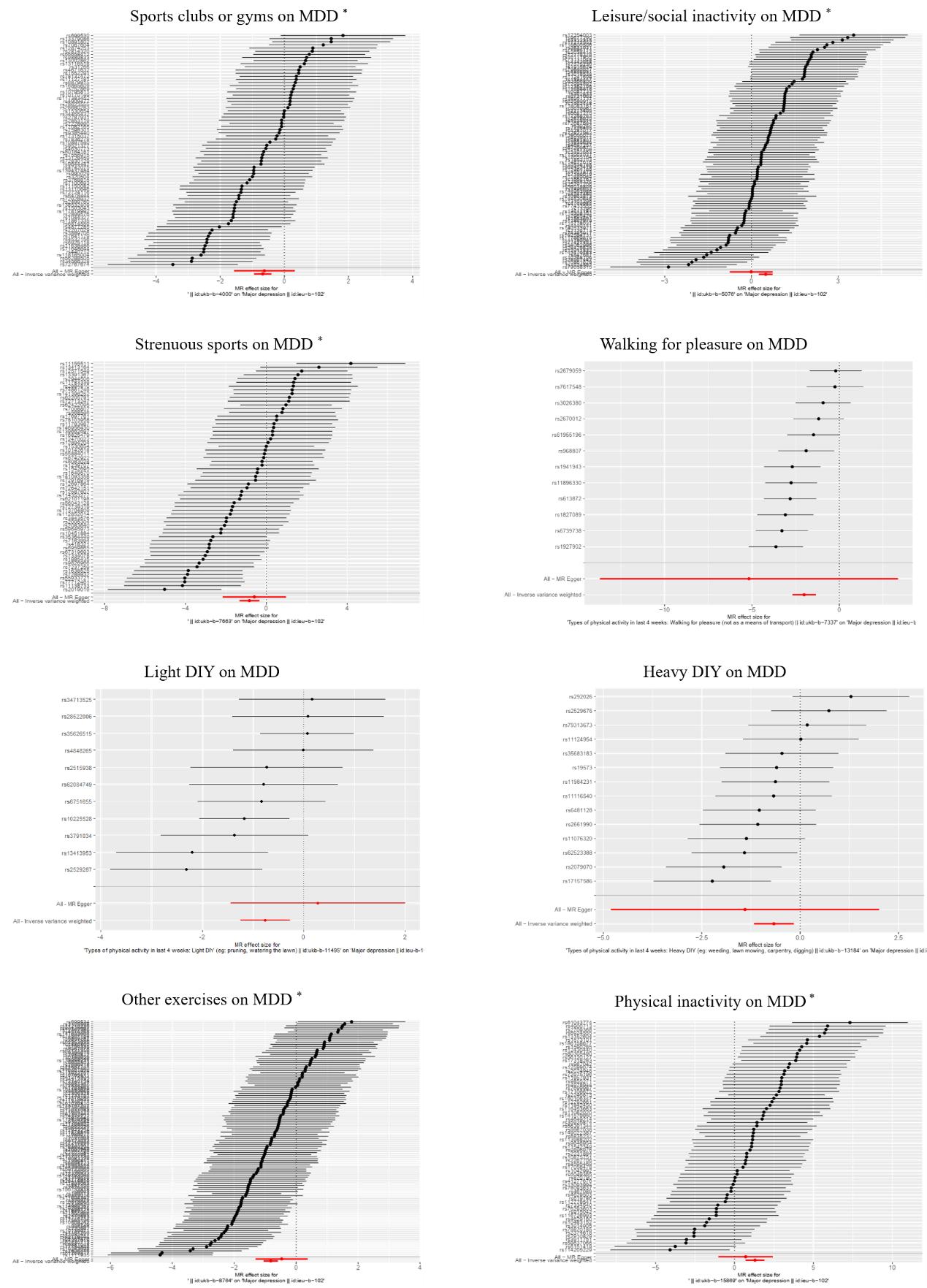


### Figure S3 The forest plots of the association between genetic predicted different activities on MDD after removing outliers

Note: ^*^ using a relaxed instrument threshold (*P*< 1×10^-5^); MDD, major depressive disorder.

### Figure S4 Leave-one-out analyses for SNPs associated with different activities on MDD after removing outliers

Note: ^*^ using a relaxed instrument threshold (*P*< 1×10^-5^); MDD, major depressive disorder.


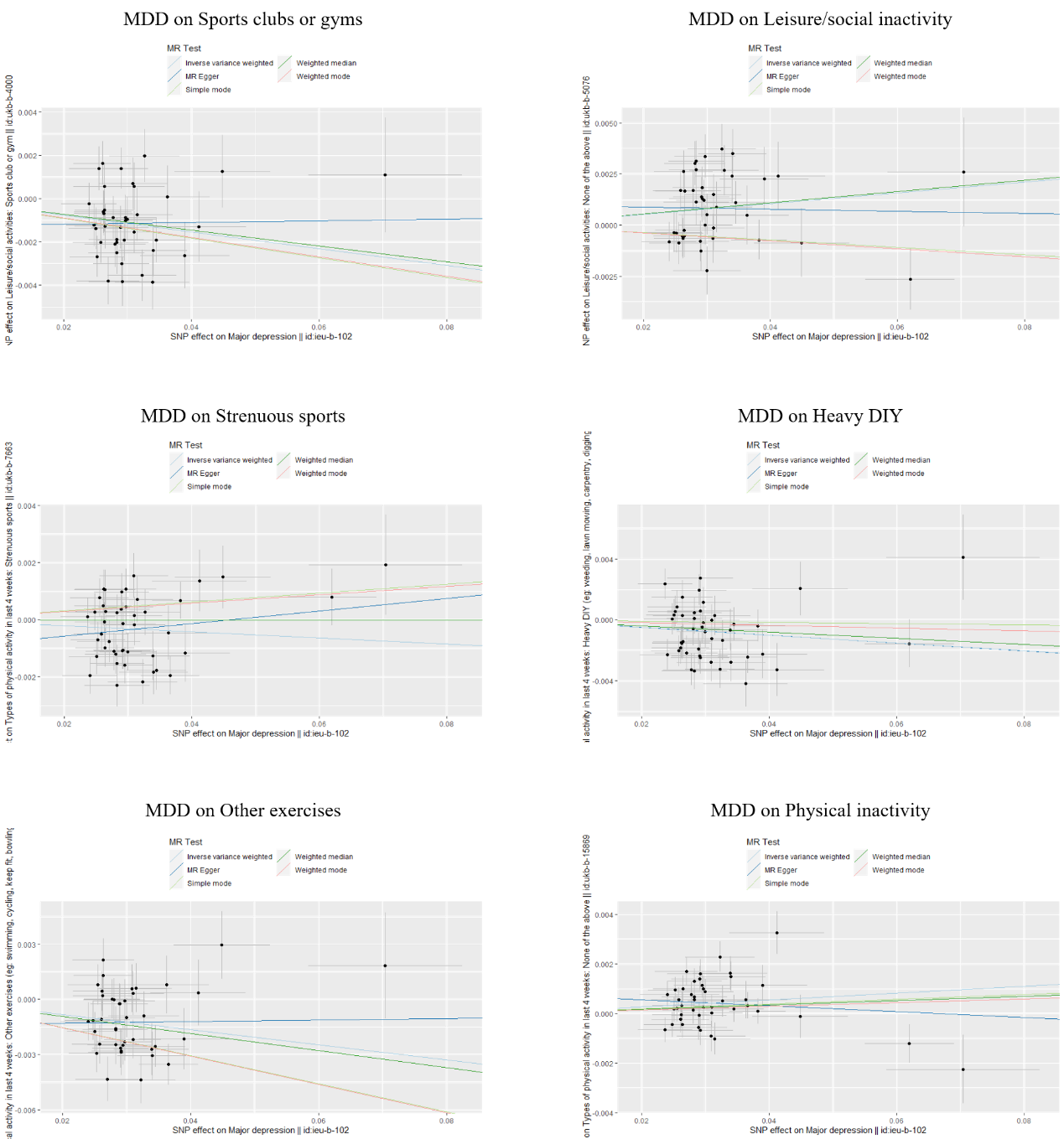


### Figure S5 The scatter plot of SNP effects on MDD versus different activities after removing outliers

Note: The slope of each line was corresponding to the estimated MR effect per method. The data are expressed as raw *β* values with 95% confidence interval. ^*^ Using a relaxed instrument threshold (*P*< 1×10^-5^); MDD, major depressive disorder.


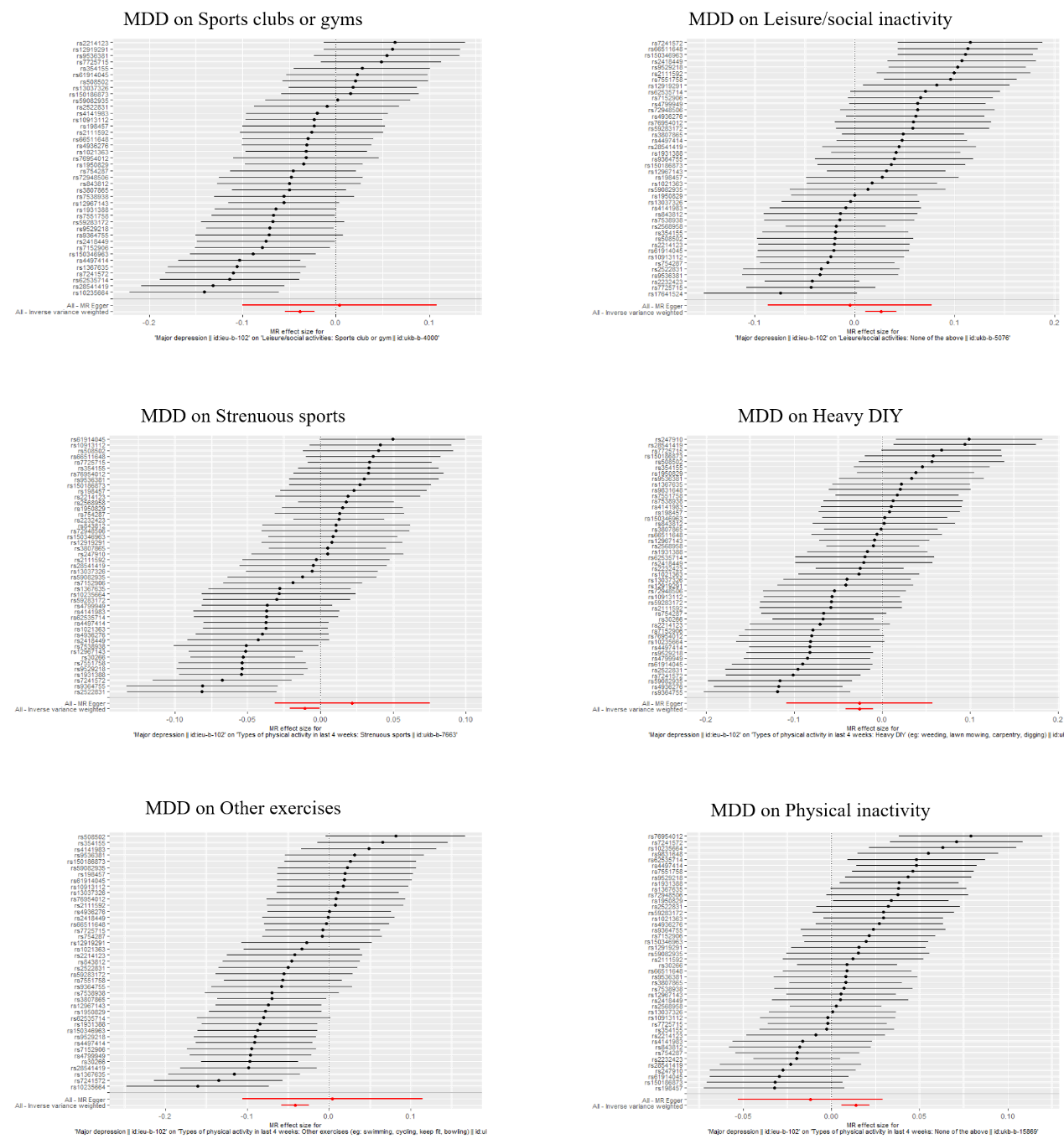


### Figure S6 The forest plots of the association between genetic predicted MDD on different activities after removing outliers

Note: ^*^ using a relaxed instrument threshold (*P*< 1×10^-5^); MDD, major depressive disorder.


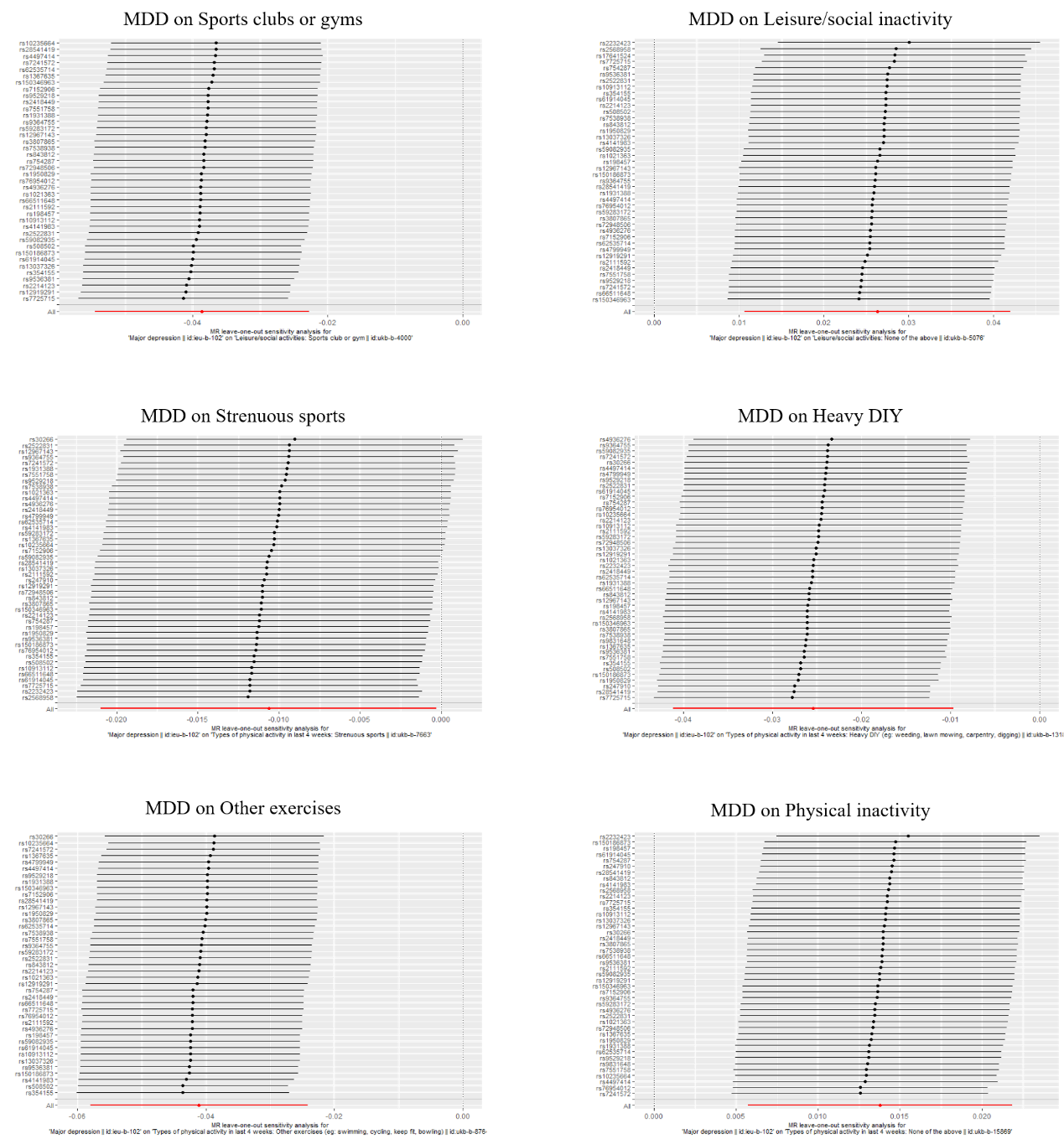


### Figure S7 Leave-one-out analyses for SNPs associated with MDD on different activities after removing outliers

Note: ^*^ using a relaxed instrument threshold (*P*< 1×10^-5^); MDD, major depressive disorder.

**References**

1. Palmer TM, Sterne JA, Harbord RM, Lawlor DA, Sheehan NA, Meng S, et al. Instrumental variable estimation of causal risk ratios and causal odds ratios in Mendelian randomization analyses. Am J Epidemiol. 2011;173(12):1392-403.

2. Zhang M, Chen J, Yin Z, Wang L, Peng L. The association between depression and metabolic syndrome and its components: a bidirectional two-sample Mendelian randomization study. Transl Psychiatry. 2021;11(1):633.

3. Hemani G, Zheng J, Elsworth B, Wade KH, Haberland V, Baird D, et al. The MR-Base platform supports systematic causal inference across the human phenome. Elife. 2018;7.

4. Bowden J, Davey Smith G, Haycock PC, Burgess S. Consistent Estimation in Mendelian Randomization with Some Invalid Instruments Using a Weighted Median Estimator. Genet Epidemiol. 2016;40(4):304-14.

5. Hemani G, Bowden J, Davey Smith G. Evaluating the potential role of pleiotropy in Mendelian randomization studies. Hum Mol Genet. 2018;27(R2):R195-r208.

6. Hartwig FP, Davey Smith G, Bowden J. Robust inference in summary data Mendelian randomization via the zero modal pleiotropy assumption. Int J Epidemiol. 2017;46(6):1985-98.

7. Mounier N, Kutalik Z. Bias correction for inverse variance weighting Mendelian randomization. Genetic epidemiology. 2023.
